# Oligodendrocyte Injury and Pathogenesis of HIV-1-Associated Neurocognitive Disorders

**DOI:** 10.3390/brainsci6030023

**Published:** 2016-07-22

**Authors:** Han Liu, Enquan Xu, Jianuo Liu, Huangui Xiong

**Affiliations:** Department of Pharmacology and Experimental Neuroscience, University of Nebraska Medical Center, Omaha, NE 68198-5880, USA; han.liu@unmc.edu (H.L.); enquan.xu@unmc.edu (E.X.); jnliu@unmc.edu (J.L.)

**Keywords:** HIV-1, dementia, oligodendrocyte, myelin sheath

## Abstract

Oligodendrocytes wrap neuronal axons to form myelin, an insulating sheath which is essential for nervous impulse conduction along axons. Axonal myelination is highly regulated by neuronal and astrocytic signals and the maintenance of myelin sheaths is a very complex process. Oligodendrocyte damage can cause axonal demyelination and neuronal injury, leading to neurological disorders. Demyelination in the cerebrum may produce cognitive impairment in a variety of neurological disorders, including human immunodeficiency virus type one (HIV-1)-associated neurocognitive disorders (HAND). Although the combined antiretroviral therapy has markedly reduced the incidence of HIV-1-associated dementia, a severe form of HAND, milder forms of HAND remain prevalent even when the peripheral viral load is well controlled. HAND manifests as a subcortical dementia with damage in the brain white matter (e.g., corpus callosum), which consists of myelinated axonal fibers. How HIV-1 brain infection causes myelin injury and resultant white matter damage is an interesting area of current HIV research. In this review, we tentatively address recent progress on oligodendrocyte dysregulation and HAND pathogenesis.

## 1. Introduction

With the introduction of combined antiretroviral therapy (cART), there was a significant decline in human immunodeficiency virus type one (HIV-1)-associated neurocognitive disorders (HAND). As HIV-1-infected patients live a longer lifespan with a cART regimen, it is becoming increasingly evident that the prevalence of milder forms of HAND seems to be on the rise [1,2,3]. Many studies have revealed a preferential damage to subcortical white matter (e.g., corpus callosum) in the HIV-1-infected brain, and such damage is prevalent even in the era of cART and more severe in patients with HAND [4,5]. HIV-1-related white matter damage includes demyelination and axonal dysfunction and injury. The demyelination occurs when myelin sheaths of neuronal axons are impaired in the central nervous system (CNS) or peripheral nervous system (PNS). Myelination, formation of myelin sheaths by oligodendrocytes wrapping neuronal axons in the CNS or Schwann cells in the PNS, is highly regulated by neuronal and astrocytic signals and maintenance of myelin sheaths is a complex process. The oligodendrocyte injury is a hallmark in demyelination and white matter damage. Such damage can be induced by an alteration of genetics, viral infections, inflammation, autoimmunity, and other unknown factors. HIV-1-associated oligodendrocyte/myelin damage has been observed both in cell culture [6] and patients [7].

The earlier studies demonstrate that human polyomavirus JC (JCV) primarily causes demyelination in HIV-1-infected brain. Compared to HIV-1 infection of astrocytes and microglia in the brain, JCV predominately infects oligodendrocytes and, thus, causes oligodendrocyte damage and further demyelination. Additionally, JCV is also the main causative factor for progressive multifocal leukoencephalopathy (PML), a frequent opportunistic infection in the CNS and a common complication seen in AIDS patients [8,7]. Recent studies have shown that HIV-1 viral proteins per se can act on oligodendrocytes and produce detrimental effects, which are independent of JCV [6,9,10]. HIV-1 viral proteins, including the envelope glycoprotein 120 (gp120), trans-activator of transcription (Tat), and negative regulatory factor (Nef), have been implicated in HIV-1-associated oligodendrocyte injury [9,11,12,13,14]. Among these viral proteins, Tat has consistently been detected in both infected and uninfected oligodendrocytes in the brains of AIDS patients [7], and exhibited a synergistic detrimental effect with JCV or with addictive drugs, such as morphine. In this review, we tentatively address recent progress in HIV-1-associated oligodendrocyte pathophysiology, aiming at understanding the pathogenesis of milder forms of HAND.

## 2. Myelin/Oligodendrocyte Injury in HIV-1 Patients

The oligodendrocyte and myelin injury have been observed clinically from neurological imaging studies, serum biochemistry, and brain biopsies [15,16,17,18]. The diffusion tensor magnetic resonance imaging (DTI) promotes the investigations of white matter damage in early HAND and allows revealing the microstructures of myelin and oligodendrocytes. The changes of water molecules’ diffusive parameters in brain white matter of HIV-1 patients, which indicate demyelination, have been detected in several DTI studies [15,19,20]. These findings were supported by a recent study on HIV-1-infected humanized mice that a decreased expression of myelin structural proteins was observed in whisker barrels, the corpus callosum, and the hippocampus, suggesting the loss of myelin elements [21]. In the sera and CSF of patients with HAND, antibody titers of myelin oligodendrocyte glycoprotein (MOG), an important myelin structural protein indicating CNS-specific autoimmune reaction for primary demyelination, are significantly higher compared with asymptomatic HAND patients and HIV-1-negative patients with other neurological diseases. In particular, the CSF anti-MOG antibodies exhibit a high sensitivity and specificity (85.7% and 76.2%) for discriminating patients with active HAND from those with asymptomatic HAND. The performance on HIV dementia scale tests is significantly worse and the viral loads in the CSF are higher in MOG immunopositive HAND patients than those in asymptomatic HAND patients [22], suggesting the dysfunction of oligodendrocytes is closely related with HIV-1 infection and HAND.

Compared to astrocytes that appear to promote recovery in response of injury, oligodendrocytes have a more passive role and tend to be damaged as a general response to insults [23]. In biopsy studies, the absolute number of nerve fibers and axons significantly decreased in HIV-1-infected brain, in particular in the frontal and occipital parts of the corpus callosum. The myelin sheath thickness diminished in corpus callosum as well [18]. Weighted gene co-expression network analysis showed that the oligodendrocyte-related genes are particularly elevated in the HIV encephalitis (HIVE) group, suggesting specific dysfunction of this cell type in those with HIVE [24].

In HIV-1 positive patients with PML, the myelin loss is apparent both macroscopically and microscopically [25]. Neuroimaging studies showed the myelin lesions were more frequently seen in the sub-cortical white matter areas [26]. PML is believed to be developed exclusively in immunosuppressive patients with significantly higher incidence in patients with AIDS, particularly in AIDS patients without cART and with a low CD4^+^ lymphocyte count, than in patients with any other immunosuppressive conditions. Although cART has decreased the incidence of PML and improved patient survival [27], PML continues to occur in HIV-1-positive patients with good access to cART, and even with normal CD4^+^ lymphocyte counts [28,29]. These findings suggest PML-related oligodendrocyte/myelin damage is often, but not necessarily, associated with severe immunosuppression or an immune reconstitution inflammatory syndrome (IRIS) in the cART era [30].

## 3. Fate of Oligodendrocytes in HIV-1-Infected Brain

Early publications reported that HIV-1 cannot be detected in oligodendrocytes [31,32] and this may due to the limitation of methodologies to identify oligodendrocytes. Dissenting results were found in purified human oligodendrocytes from temporal lobe resections, HIV-1 (IIIB and BaL) infectivity was confirmed by detection of p24gag antigen and PCR amplification [33]. It is well-known that HIV-1 attaches and infects human host cells through CD4 receptors, along with CXCR4 and CCR5 as co-receptors. The oligodendrocytes are CD4- and CCR5-negative, but do express CXCR4 [31,34,35], which designedly promote the oligodendrocyte progenitor cell (OPC) migration and remyelination [36], and may provide the anchor for HIV-1-induced oligodendrocyte injury. However, most investigators agree that HIV-1 primarily infects microglial cells in the brain, but not oligodendrocytes. HIV-1-associated oligodendrocyte injury is believed to be mediated through viral proteins shed off from virions or released from infected other cells [9,11,12].

In HIV-1 patients with PML complication, Tat and JCV both are present in oligodendrocytes. Tat has been shown to synergize with JCV, and facilitate of JCV gene transcription and replication, leading to robust JCV infection [37,38]. Tat stimulates JCV gene transcription by cooperating with SMAD proteins, the intracellular effectors of TGF-beta, at the JCV DNA control region [37]. The effectiveness of Tat on facilitating JCV transcription and replication varies from different HIV-1 clades [38]. Since Tat is expressed in the brain at relative high levels while the viral load is controlled in blood, this may, at least in part, explain why some HIV-1 patients still develop PML despite having a good access to cART [39]. In addition to the synergistic effect of Tat and JCV in oligodendrocytes, cytotoxic CD8^+^ T cells aggregate at demyelinated lesion sites in the brain to engage JCV-infected oligodendrocytes, which tend to control JCV dissemination, but at the cost of oligodendrocyte death and further demyelination in PML [40].

In addition to Tat, gp120 seems to be also involved in HIV-1-associated oligodendrocytes/myelin injury. It has been shown that gp120 inhibits myelination in rat cerebral cortex culture [12] and induces functional dysregulation and apoptosis in cultured oligodendrocytes [11,41], which is discussed in a subsequent section. In addition to the primary oligodendrocyte injury, which leads to secondary axonal injury (outside-in) to further exacerbate neurocognitive impairments, oligodendrocyte injury can be caused by primary axonopathy as well (inside-out) [42]. The recent study has shown that gp120-induced β-APP accumulation and axon injury in the corpus callosum was attenuated by a CXCR4 antagonist, exampling HIV-1 injury of oligodendrocyte/myelin via CXCR4 [35]. Although it is not clear whether gp120 causes such a detrimental effect through an “outside-in” or “inside-out” mechanism, or both, CXCR4 expressed in oligodendrocytes can be a potential target [42].

## 4. Association between Blood-Brain Barrier (BBB) Disruption and Myelin Injury

Increasing evidence indicates that myelin injury may be associated with a dysregulated blood-brain barrier (BBB) since myelin pallor is often observed in perivascular sectors during white matter edema [43,44]. The BBB is a critically-protective barrier for the brain and serves as a highly selective layer that separates the CNS from the rest of the body. In HIV-1-infected brains, the BBB disruption is believed to be mediated by both viral and cellular factors, released from HIV-1-infected and immune-activated mononuclear phagocytes and endothelial cells [45,46]. The reported direct mechanisms underlying HIV-1-associated BBB disruption are often related to alterations of vascular tight junctions, direct toxicity of brain endothelial cells, production of matrix metalloproteinases, and N-Methyl-D-Aspartate (NMDA) receptor activation [47,48]. The disruption of BBB is essential for HIV-1 entrance to the brain, resulting in brain white matter damage and consequent neurologic deficits in patients with neuroHIV. This notion is supported by an observation that HIV-1 patients with impaired BBB showed poorer neurologic status than those with intact BBB [49]. However, myelin damage may also be related to BBB disruption without HIV-1 brain invasion. In a case report, diffuse myelin pallor in white matter and massive perivascular dilatation were observed in an AIDS patient without evidence of brain HIV-1 infection, significant inflammation, or microglial activation [50]. Postmortem studies on the brains of AIDS patients revealed discrete myelin pallor areas always associated with capillaries or venules [44]. These findings suggest that BBB breakdown may contribute to the observed oligodendrocyte/myelin/white matter injury.

Under physiological conditions, the BBB endothelial cells and components of the extracellular matrix support OPC survival, and promote neural progenitor cells (NPC) differentiate to neurons, astrocytes, and oligodendrocytes [51,52,53]. The critical function of BBB and the consequences of BBB disruption imply its involvement in HIV-1-associated oligodendrocyte/myelin injury. It is not surprising that the disrupted BBB promotes the entrance of viruses, infected T cells, and toxic substances from the blood to the brain, resulting in OPC/oligodendrocyte/myelin injury. Inhibition of OPC proliferation can be caused by plasma, serum, thrombin, and plasmin in primary culture. Thrombin also suppresses the differentiation of OPCs into mature oligodendrocytes [54]. In addition, elevated levels of TNF-α, an inflammatory cytokine promoting oligodendrocyte death [55], were detected in blood mononuclear phagocytes in HIV-infected patients [56]. HIV-1 infection also induces interleukin (IL)-1β production from mononuclear phagocytes [57]. IL-1β promotes oligodendrocyte death through glutamate excitotoxicity [58]. These findings suggest that BBB disruption contributes to HIV-1-associated myelin/oligodendrocyte damage.

## 5. Cellular Mechanisms for Oligodendrocyte Injury in HIV-1-Infected Brain

Apoptotic signal activation of oligodendrocytes has been observed in HAND patients [59]. Such an apoptotic activation of oligodendrocytes could be caused directly by HIV-1 viral proteins or induced indirectly by immune and inflammatory factors.

It is well-known that tumor suppressor p53 induces apoptosis by activating transcription of various pro-apoptotic genes [60]. Activation of p53 was detected in the oligodendrocyte lineage cells in the brains of HAND patients, but not in control brains [59]. Due to the difficulties in distinguishing the differentiating stages of oligodendrocyte lineage on autopsy samples, the detected p53 reactivity reflects apoptosis of mature oligodendrocytes and OPCs. These suggest that, in addition to oligodendrocyte injury, proliferation of OPCs is also impaired in HAND.

Gp120 was shown to cause slow but progressive oligodendrocyte cytosolic Ca^2+^ rise in a mixed culture of cerebellar cortex cells [41] and the rise of introcellular Ca^2+^ concentration may trigger oligodendrocytic apoptosis. Exposure of oligodendrocytes to Tat also produced a rapid increase in intracellular Ca^2+^ levels through NMDA and α-amino-3-hydroxy-5-methyl-4-isoxazolepropionic acid (AMPA) receptors, causing oligodendrocyte injury. It is worth mentioning that the roles of NMDA and AMPA receptors appear to be different and dependent on the stage of OPC differentiation. Tat-induced OPC death can be blocked by either NMDA or AMPA receptor antagonists. However, Tat’s detrimental effects on mature oligodendrocytes can only be reversed by NMDA receptor antagonists, but not AMPA receptor blockade [6]. Tat was also found to cause oligodendrocyte apoptosis *in vitro* and myelin injury *ex vivo* by enhancing voltage-dependent K^+^ channel (Kv) 1.3 activity [61]. The loss of K^+^ ions may cause cell regulatory volume decrease (shrinkage), leading to cell apoptosis [62]. The involvement of Tat in oligodendrocyte apoptosis has been demonstrated in an HIV-1 Tat transgenic mouse model. The oligodendrocytes within the striatum exhibit a high sensitivity to morphine in HIV-1 Tat transgenic mice and they are the only apoptotic cell type in response to combined morphine exposure and Tat induction in Tat transgenic mice [9]. Tat also interacts with morphine to decrease the proliferation of OPCs [63]. Opioid abuse produces synergistic toxic activity in HIV-1-infected brains by direct actions on immature astrocytes and oligodendrocytes, which express µ-opioid or κ-opioid receptors [64].

In other viral-induced demyelination, there is clear evidence that mouse hepatitis virus (MHV) can directly infect and activate microglia during acute inflammation, which eventually causes phagocytosis of the myelin sheath, leading to demyelination during the chronic inflammation stage [65]. A similar theory has been proposed for multiple sclerosis, which is the most prevalent demyelinating disease, that immune-activated microglia strip the myelin. Recent evidence has shown that microglia become phagocytic in response to HIV-1 Tat [66,67]. It might be possible that the infected and activated microglia phagocyte oligodendrocytes and myelin sheath lead to the myelin damage and consequent HAND pathogenesis, although there is no direct evidence indicating microglia phagocytosis of oligodendrocyte in neuroHIV [68]. 

## 6. Myelin Maintenance and Remyelination in HIV-1-Infected Brain

Repair of the damaged myelin sheath, which is termed remyelination, is physiologically required to maintain myelin homeostasis. The myelin injury in neuroHIV may also be induced by abnormalities of remyelination, in addition to the loss of existing myelin sheath. Remyelination requires proliferation and survival of OPCs, migration of OPCs to the damaged site, and development of OPCs from immature to mature myelinating oligodendrocytes. HIV-1 disrupts OPC development, migration, and remyelination processes.

### 6.1. Alteration of OPC Proliferation and Differentiation in HIV-1-Infected Brains

In HIV-1-infected brains, mild degrees of myelin damage were associated with an increase in oligodendrocyte numbers, an initial reactive hyperplasia which was believed to represent an attempt to repair myelin damage. Such a change was reversed in the presence of severe myelin damage [69]. In agreement with the aforementioned results, mRNA levels of transcription factor Olig2, a marker expressed with higher levels in OPCs and lower levels in mature oligodendrocytes [70], are elevated in the front cortex of patients with HIVE [24], indicating an increase of OPC proliferation needed for repairing the damaged myelin sheath. Mature oligodendrocyte defects are also observed in animal models of secondary degeneration, which represents additional loss of neurons, myelin, and glial cells through toxic events. Early onset of secondary degeneration triggers OPC proliferation, but the cell numbers decrease in a long-term degenerative condition [71]. However, Tat exposure reduces the population of undifferentiated Sox2^+^ NPC (ancestor of OPC) and Olig2^+^ OPCs, but progenitor survival is unaffected [63], suggesting the proliferation was interrupted. Tat may inhibit NPC proliferation by downregulating cyclin D1, which is an important cell cycle component interacts with cyclin-dependent kinase 4 and 6 [72]. Over all, HIV-1 infection or viral protein exposure appears to incline NPC fate toward production of glia/astroglia at the expense of neurons and/or oligodendrocytes [63,73,74]. Thus, OPC differentiation and maturation are likely the key processes affected during remyelination in neuroHIV.

### 6.2. Imbalance of OPC Differentiation and Remyelination in HIV-1-Infected Brain

It has been shown that differentiation of OPCs into post-mitotic oligodendrocytes is a major checkpoint in the myelination process and such an oligodendrocyte differentiation is controlled by a number of factors, many of which act to inhibit myelination, including leucine-rich repeat and immunoglobulin domain-containing-1 (LINGO-1) [75,76], Notch-1 [77,78], and Wnt [79,80], whereas p38 MAPK [81,82] and AKT [83] have been shown to be required for oligodendrocyte differentiation and myelination. While molecular mechanisms in the regulation of developmental myelination are discussed in excellent review papers published elsewhere [84,85], the direct interaction between HIV-1 and these molecules remains largely unknown. It is, however, believed that HIV-1 may disturb the complex regulating network leading to the remyelination imbalance based on the following findings: (1) HIV-1 alters the cell cycle by Wnt signaling pathways and further impacts the cell proliferation and differentiation in different cell types, including peripheral blood mononuclear cells [86], HEK293 cells [87], and astrocytes [88]; (2) HIV-1 infection of astrocytes altered the astrocytic Wnt profile by elevating Wnt family members 2b and 10b [88]; (3) elevation of secreted Wnt from astrocytes may negatively regulate oligodendrocyte differentiation in neuroHIV; (4) Notch-1 signaling is permissive for OPC expansion, but inhibit differentiation and myelin formation [89]; and (5) in Kaposi’s sarcoma cells, which is a neoplasm in HIV-1-infected individuals, overexpression of activated Notch-1 signaling is detected [90]. However, to our knowledge, there is no report yet on how OPC/oligodendrocyte Notch-1 signaling in responding to HIV-1. Moreover, CD44, a predominant hyaluronan receptor widely expressed in the nervous system, plays a negative role in OPC differentiation and myelination. Overexpression of CD44 in precursor cells inhibits differentiation toward oligodendrocytes and promotes differentiation into astrocytes, cause progressive demyelination in conditional transgenic mouse model [91,92]. In lymphocyte cell lines of Jurkat and U937 cells, HIV-1 infection-caused particle production is accompanied by CD44 upregulation [93]. In HIV-1-related diffuse large B-cell lymphoma patients, the CD44 levels significantly increased compared with HIV-1-unrelated diffuse large B-cell lymphoma patients (87% vs. 56%) [94]. These findings suggest CD44 may play a role in HIV-1-related remyelination failure.

Neurotrophins are important factors in the regulation of oligodendrocyte myelination and remyelination. The main cellular sources for neurotrophins in the brain are astrocytes, microglial cells, and neurons, in addition to lymphocytes’ contribution through the blood circulation. Being aware of excellent reviews on the alteration of neurotrophins in HAND [95] and immunological communications between oligodendrocytes and microglia [96], we focus here on the HIV-1-induced alterations of neurotrophins that are potentially associated with oligodendrocyte abnormalities.

The platelet-derived growth factor (PDGF) is the most predominant mitogen for oligodendrocyte lineage cells. PDGF A and B chains both promote proliferation through activating PDGF receptor alpha (PDGFRα) expressed on OPCs, whereas the PDGF B chain appears to be more important for early NPC expansion [97,98]. It has been shown that PDGF regulates OPC development via glycogen synthase kinase-3β (GSK-3β) signaling pathway, which is a negative regulator of OPC differentiation and remyelination [99,100]. PDGF-BB prevents NPC from Tat-mediated proliferating impairment by inactivating GSK-3β/β-catenin pathways and, this effect is significantly inhibited by the p38 and JNK inhibitors [101]. The levels of fibroblast growth factor (FGF), which is an important pro-survival signal to stimulate OPC proliferation [102], increased in the sera of HIV-1-infected patients [103,104], but decreased in CSF [103]. FGF signaling complex is interrupted in HIV-1-infected brains, resulting in the abnormal activation of downstream signals, including GSK-3β [105,106], p38, ERK, and JNK cascades [107] in neurons through the surface receptors, such as NMDA receptor and CXCR4, which are also expressed on oligodendrocytes [36,108,109]. In addition, HIV-1 Tat and FGF-2 share a common core mechanism of unconventional secretion [110], although it is not clear whether they compete for the secretory routine. The brain-derived neurotrophic factor (BDNF), predominantly derived from astrocytes, has also been found to be essential for oligodendrocyte lineage development [68,111,112,113]. In rat primary neurons, gp120 promotes a time-dependent proBDNF accumulation at both intracellular and extracellular spaces by decreasing the expression level of intracellular furin, an enzyme required for cleavage and release of mature BDNF, leading to a reduction in mature BDNF. A similar imbalance in the ratio of proBDNF/mature BDNF was confirmed in postmortem brains of HAND patients [114]. These findings suggest that HIV-1 decreases the brain BDNF level by infecting astrocytes and gp120-associated neurotoxicity, resulting in downregulated remyelination. As BDNF is believed to protect neurons from HIV-1-induced apoptosis, thus, the reduction of BDNF may make the oligodendrocyte lose the support from neuronal axons that consequently cause myelin damage through the “inside-out” mechanism as proposed [42].

In addition to these signaling molecules, HIV-1 Tat interacting protein (TIP30), a co-factor that specifically enhances HIV-1 Tat-activated transcription [115], negatively regulates oligodendrocyte development. Overexpression of TIP30 dramatically inhibits the OPC differentiation, while knockdown of TIP30 enhances the differentiation of OPC remarkably [116]. The blockade of TIP30 may have dual benefits on inhibiting Tat-dependent gene transcription and promoting OPC differentiation, which is a potential therapeutic strategy for HIV-associated demyelination. Potassium channels are also involved in regulation of OPC development. Kv1.3 [117,118], Kv1.6 [117], Kv2.1 [119], and inward-rectified K^+^ channel 4.1 [120,121] play crucial roles in regulation of OPC/oligodendrocyte proliferation and differentiation. Generally, channel expressions on oligodendrocyte lineage cells correlate with differentiating stages and are more complex in OPCs than in oligodendrocytes. Particularly, Kv1.3 channel plays an important role in G1/S transition in proliferating OPCs through regulating AKT signaling [118,122]. Moreover, L-type voltage-operated Ca^2+^ channel 1.2 knockdown induces a decrease in the proportion of oligodendrocytes expressing myelin proteins, and an increase in the population of immature oligodendrocyte [123].

Most recent studies proposed that myelin injury in HAND is partially due to the effects of antiretroviral drugs on oligodendrocyte survival and differentiation. The common prescribed antiretroviral drugs, ritonavir and lopinavir, impair both the differentiation of OPCs into myelin-producing oligodendrocytes and the maintenance of myelin proteins *in vivo*. Ritonavir induces accumulation of reactive oxygen species, which arrest the oligodendrocyte differentiation process [124,125]. Controversial results were reported in HIV-1-infected children in Africa that significant myelin loss in cART-naïve children was observed in comparison with cART-treated children. However, cART-treated children also exhibited a significant myelin loss in the corpus callosum [126]. Interestingly, myelin-related genes encoding myelin-associated oligodendrocyte basic protein, myelin transcription factor 1, and myelin basic protein are downregulated in both cART-treated and untreated HAND patients [127]. Apparently, the impact of antiretroviral drugs on oligodendrocyte pathophysiology requires further investigation.

## 7. Summary and Prospects

HIV-1 persists in the brain despite cART. The cART-treated subjects are not able to purge the virus from their brains and show concomitant and persistent white matter abnormalities. There are increasing interests in understanding how HIV-1 causes myelin sheath loss and white matter damage in HIV-1-infected brains. In this article, we try to address the clinical and postmortem manifestations of myelin damage in HAND patients and possible involvement of BBB integrity disruption, oligodendrocyte apoptosis mechanisms, and OPC regulation imbalance in HIV-1-induced oligodendrocyte/myelin abnormalities. The studies on direct toxicity of HIV-1 viral proteins on oligodendrocytes and OPCs are emerging. As the transcription of HIV-1 viral protein continues in the CNS, even when the viral load is at a low level [128], the persistence of the virus and viral proteins in the brain has changed the pattern of HAND pathogenesis, by which inflammation, encephalitis, and neurodegeneration have been significantly decreased by the advent of cART.

The methods of regulation of oligodendrocyte lineage cell development are well-established, including the extracellular pathways, cell to cell contact, and intracellular pathways. As NG2^+^ cells are the largest population of progenitor cells in the human adult brain, a decrease of absolute cell number and proliferation of NPC and OPC may contribute less to myelin deficits in HAND. In contrast, HIV-1-related OPC differentiation and remyelination imbalance may better correlate with an impaired remyelination in HAND patients. The strategies for promoting axonal remyelination have been introduced especially in those demyelinating disease like multiple sclerosis. It is anticipated that those strategies for promoting axonal remyelination in other neurodegenerative disorders can be applied for HIV-1-associated oligodendrocyte/myelin injury, though studies are needed to elucidate the underlying mechanisms for HIV-1-associated brain white matter damage.

Further studies on understanding the mechanisms underlying HIV-1-associated oligodendrocyte/myelin injury may be hampered by the following potential difficulties: first, oligodendrocytes share many common extracellular signals and intracellular signaling pathways with neurons, the proposed “inside-out” and “outside-in” mechanisms for virus-induced demyelination are indistinguishable under these conditions [42]; and, second, the pro-proliferation signals for OPC are sometimes anti-maturative [129,130,131]. This will be a significant challenge to identify the certain time window to access proper remyelination in vivo. Overall, promoting remyelination could be an important therapeutic strategy for HAND and other neurodegenerative disorders in the future.
